# Crowning: a novel *Escherichia coli* colonizing behaviour generating a self-organized corona

**DOI:** 10.1186/1756-0500-7-108

**Published:** 2014-02-25

**Authors:** José María Gómez-Gómez, Ricardo Amils

**Affiliations:** 1 Departamento de Virología y Microbiología, Laboratorio 104. Centro de Biología, Molecular Severo Ochoa, Campus of the Universidad, Autónoma de Madrid, C/ Nicolás Cabrera n° 1, Madrid 28049, Spain; 2 OAS-BioAstronomy Group, Observatorio Astronómico de Segurilla (OAS), 45621, Camino de Valparaiso S/N, Segurilla Toledo, Spain; 3 Centro de Astrobiología (CSIC-INTA), (Associated with NASA Astrobiology Institute), Ctra Ajalvir km 4, Torrejón de Ardoz, Madrid 28850, Spain

**Keywords:** Biofilms, *Escherichia coli*’s corona, Crowning behaviour, Macrocolony biofilm, CsgD-independent behaviour, Glucose repression, CRP-independent behaviour

## Abstract

**Background:**

Encased in a matrix of extracellular polymeric substances (EPS) composed of flagella, adhesins, amyloid fibers (curli), and exopolysaccharides (cellulose, β-1,6-*N*-acetyl-D-glucosamine polymer-PGA-, colanic acid), the bacteria *Escherichia coli* is able to attach to and colonize different types of biotic and abiotic surfaces forming biofilms and colonies of intricate morphological architectures. Many of the biological aspects that underlie the generation and development of these *E. coli*’s formations are largely poorly understood.

**Results:**

Here, we report the characterization of a novel *E. coli* sessile behaviour termed "crowning" due to the bacterial generation of a new 3-D architectural pattern: a corona. This bacterial pattern is formed by joining bush-like multilayered "coronal flares or spikes" arranged in a ring, which self-organize through the growth, self-clumping and massive self-aggregation of cells tightly interacting inside semisolid agar on plastic surfaces. Remarkably, the corona’s formation is developed independently of the adhesiveness of the major components of *E. coli*’s EPS matrix, the function of chemotaxis sensory system, type 1 pili and the biofilm master regulator CsgD, but its formation is suppressed by flagella-driven motility and glucose. Intriguingly, this glucose effect on the corona development is not mediated by the classical catabolic repression system, the cyclic AMP (cAMP)-cAMP receptor protein (CRP) complex. Thus, corona formation departs from the canonical regulatory transcriptional core that controls biofilm formation in *E. coli*.

**Conclusions:**

With this novel "crowning" activity, *E. coli* expands its repertoire of colonizing collective behaviours to explore, invade and exploit environments whose critical viscosities impede flagella driven-motility.

## Background

Bacteria were traditionally considered single-cellular forms of life. In the last few years a Kuhnian paradigm shift has brought us to the realization that bacteria, on the contrary, are gregarious organisms enjoying an intense array of social activities and living in complex multicellular communities [1,2]. Thus, sociability, i.e., bacteria as highly communicated and organized societies, and multicellularity, i.e., different kinds of phenotypically differentiated (with different functions) sibling cells working together as a unique organism to reach a "common goal" of survival and organismal reproduction, represent the new conceptual framework with which to understand the microbial world [3-7]. A bacterial multicellular formation that allows this realization to be clearly understood is the biofilm [8,9].

Biofilms are defined as matrix-enclosed communities of microorganisms tightly interacting with each other, attached as a whole to a living or non-living surface [9,10]. In *Escherichia coli* the extracellular matrix is formed by a variety of extracellular polymeric substances (EPS), adhesions: type-1 pili [11], amyloid fibers (curli) [12] and exopolysaccharides: cellulose [13], colanic acid [11,12], β-1,6-*N*-acetyl-D-glucosamine polymer (PGA) [14,15] (reviewed in reference [16]). Bacterial flagella have a positive role in nascent biofilms, facilitating initial attachment to surfaces, maturation and microcolony spreading [11] as well as carrying out a structural role [17]. Interestingly, it has been described that the type-1 pili and the outer membrane Ag43 are two factors that influence the colony morphology of certain *E. coli* K-12 strains [18]. Mature biofilms form complex three-dimensional architectures, described typically mushroom-like forms, exhibiting channels and pillars that may facilitate nutrient exchange and waste removal [8]. The biofilm is a sessile lifestyle that provides bacteria with multiple protective advantages by protecting them from different kinds of external stress: antibiotic, osmotic, temperature, acidity, oxidative, heavy metals, desiccation and predators [9]. These phenotypic traits pose a challenge to the eradication of persistent infections [19].

Like most bacteria, *E. coli* can switch between being motile nomadic (using their peritrichous flagella) wandering planktonic single-cells when searching for nutrients and favourable environments, i.e., a "foraging stategy" [20] (chemotactically guided, [21]), to living embedded in a biofilm [16]. Underlying these choices is a complex transcriptional regulatory network that controls the switching between states [22]. This switch mechanism is implemented by two inversely controlled transcriptional feedforward cascades, the FlhDC + σ^70^/σ^F^ "flagellar" cascade for the expression of genes involved in flagellum synthesis and operation, chemotaxis, and related functions in a three-layer cascade [23] operation that drives the cell to a planktonic motile mode and promotes collective flagella-driven bacterial movement on semisolid surfaces (swarming motility) [24-27] and the σ^S^/MlrA/CsgD cascade for control and surface adhesiveness reviewed in reference [28].

In the σ^S^/MlrA/CsgD cascade, the transcriptional master biofilm regulator CsgD (a transcriptional activator belonging to the FixJ subfamily of two-component response regulators [29,30]) acts promoting sessility through the activation of expression of the *csgBAC* and *csgDEFG* curli operons, which encode the structural genes for synthesis, secretion, and assembly of adhesive curli fimbriae and indirectly activates cellulose biosynthesis [30].

It is important to the logic of the switch mechanism that both cascades show mutual reciprocal inhibition. Thus, FlhDC + σ^70^/σ^F^ "flagellar" cascade downregulates the σ^S^/MlrA/CsgD cascade expression by reducing the transcription of a subset of σ^S^-dependent genes mediated by the regulator FliZ (a flagellar class II gene) [22] and by maintaining low levels of c-di-GMP *via* YhjH, an EAL protein, and c-di-GMP phosphodiesterase (PDE) activity under FlhDC/FliA control [22], preventing the inhibition of flagella motor function by YcgR, a PilZ domain protein that is activated upon c-di-GMP binding [31]. Inversely, CsgD represses the expression of genes related to flagellum formation, assembly [30] and rotation inhibiting cell motility [30] and also interferes with flagellar motor speed through the YcgR protein [32].

Recently, a "pleyade" of small RNA (sRNA) has been unveiled working in this regulatory network in *E. coli* that are responsible for fine-tuning the FlhDC and CsgD expressions to different environmental cues [28,33-35].

Carbon metabolisms play an important part in biofilm formation [36]. Catabolite repression is the preferential utilization of glucose as a carbon source by bacteria [37,38]. When glucose is available, uptake and utilization of alternative carbon sources are repressed (i.e., catabolic repression). It is well known that catabolite repression plays an important role in the regulation of multilayer biofilm formation in many bacteria [36]. For instance, glucose represses the biofilm formation in several species of *Enterobacteriaceae* and laboratory strains of *E. coli*[39]*.* Catabolite repression in *E.coli* biofilm formation has been reported to be mediated in part by cyclic AMP (cAMP) and the cAMP receptor protein (CRP) [39].

However, despite the notable advances in clarifying the physiology and regulatory circuitry that allow *E. coli* bacteria to transit adaptively among these different lifestyles, many aspects of how these microorganisms live in these diverse bacterial cities [40] as well as how the 3-D biofilm morphogenesis is spatially organized and generated are largely unknown.

Given the important role of biofilms in bacterial pathogenesis, we sought to study the *E. coli* behaviour under environmental conditions that have not been previously considered. In this paper, we describe a previously unknown colonizing behaviour of *E. coli* observed in old colonies.

## Results and discussion

**Figure 1:**
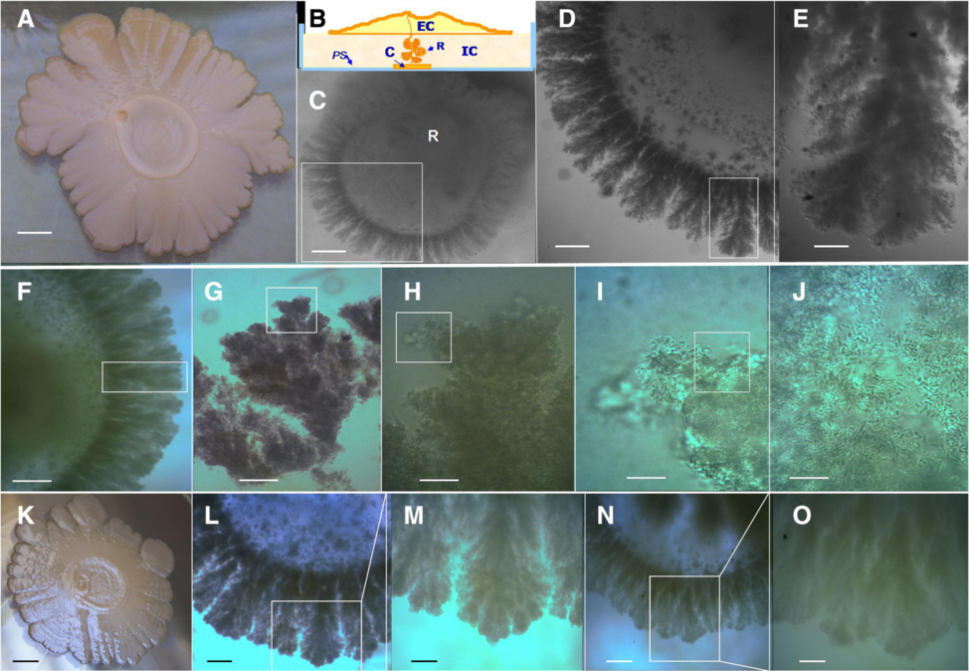
**Old macrocolony biofilms are producers of *E. coli’s *corona. (A)** Typical volcano-like appearance (morphotype) of a 14-old-day macrocolony biofilm of *E. coli* K-12 MG1655 strain developed over a semisolid 0.6% ABE agar surface visualized by reflected light. **(B)** Schematic representation of characteristic structures observed in this macrocolony. EC, *external colony* corresponds to the superficial aerial, visible part of macrocolony developed on the semisolid agar surface. IB, represent the *internal biofilm,* an agar-entrapped formation, composed of "root" (R) and "corona" (C) developed inside semisolid agar. The root is the structure generated by bacterial "fingers" that penetrate inside the agar along the toothpick-punctured zone, while the corona (C) is the biofilm structure that develops in contact with the plastic surface (PS) of the base of the Petri dish. **(C)** Typical 14-old-day corona of *E. coli* K-12 MG1655 strain. **(D-E)** Close-up of the corona and a coronal spike. **(F-J)** Zooming view of a coronal spike removed from a corona and placed under a microscope at different magnifications. **(F)** Part of a corona viewed to × 40, the box indicates the coronal spike removed and observed at different magnifications: **(G)** × 100 **(H)** × 400 **(I-J)** ×1000. **(K)** Typical 14-old-day *E. coli* K-12 BW25113 strain volcano-like colony and their corona **(L-M)**, (L) × 40 (M) × 100 **(N-O)**. Typical 14-old-day corona of *E. coli* K-12 W3110 strain. (N) × 40 (O) × 100 magnifications. Each box (right) represents the enlargement region in the following image (left). Scale bars: (A, K) 0.5 cm (C) 0.1 cm (D) 500 μm (E, M, O) 100 μm (F) 350 μm (G) 150 μm (L, N) 200 μm (H) 40 μm (I-J) 20 μm.

**Figure 2:**
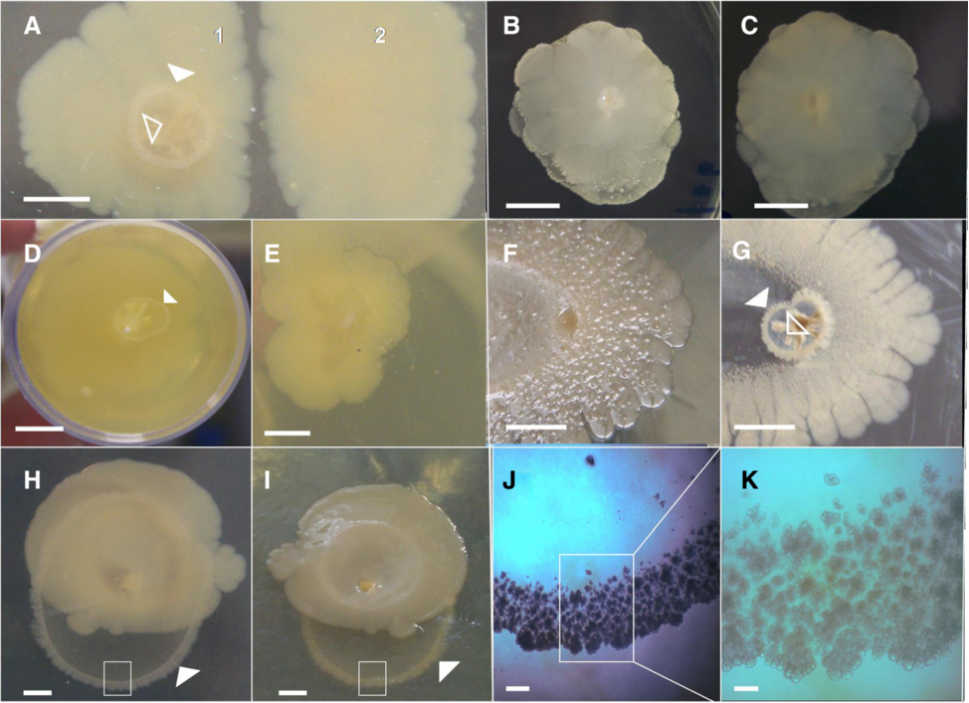
**Environmental and biological factors that promote the *E. coli *K-12 corona formation or else preclude this formation. (A)** The colony (A-1) produced the *E. coli*’s corona when it was inoculated with a toothpick that punctured the 0.6% ABE semisolid agar and contacted the Petri dish’s plastic surface. The colony (A-2) by contrast did not generate a corona when the bacterial inoculation was carried out with a drop containing 5 μl of a stationary culture grown in LB medium of MG1655 strain deposited carefully on semisolid agar surface **(B-C)** The flagella-driven swimming interstitial/internal motility abolished the corona formation. Typical 14-old-day macrocolony swimming colony of *E. coli* K-12 MG1655 strain **(B)** View from the top **(C)** Reverse view **(D)** Corona developed on a plastic surface, but not on a glass surface **(E)**. **(F)** A typical 14-old-day macrocolony of *E.coli* K-12 W3110 strain **(G)** Appearance of the same colony when the external colony was removed **(H-I)***E. coli*’s corona developed inside semisolid 1.0% ABE agar concentration. **(H)** View from the top **(I)** Reverse view. **(J-K)** The enlarged box region in (H-I) showing the corona at × 40 **(J)** and ×100 **(K)** magnifications. The closed white arrowheads indicate the situation of corona. The open white arrowheads indicated the coronal root. Scale bars: (A-G), 0.5 cm; (H-I) 0.25 cm; (J) 300 μm; (K) 100 μm.

The genetic determinants and environmental cues that impact on pattern formation in *E. coli* old-macrocolony biolfilms are poorly understood. To learn more about this subject we initially use the wild-type *E. coli* K-12 MG1655 strain. Figure 1A shows a typical 14-day-old macrocolony of this strain developed on semisolid agar surfaces under the growth conditions defined for this study (see Methods) exhibiting a volcano-like colonial morphotype. Surprisingly, when we carried out a visual inspection of the reverse side of the Petri dish (Figure 1B) we observed a striking bacterial formation that developed in contact with the plastic surface of the bottom of the plate within the semisolid agar: a corona (Figure 1C). Similar colonial morphotypes and coronal patterns were observed with other *E. coli* wild-type strains: BW25113 (Figure 1K-M), W3110 (Figure 1N-O and Figure 2F-G) and NM525 (data not shown). To our knowledge, the pattern of both *E. coli* biofilm formations, both volcano-like and corona have not been previously reported. In this report, we described the structural organization and the environmental, nutritional and genetic factors that affect corona development.

A typical 14-day-old completely developed corona is approximately 0.6 cm in diameter (Figure 1B) and is formed by adjoining, annularly arranged arrays of multilayered pillars, which constitute the "coronal flares" or "spikes" of the corona (Figure 1C-E). These structures, showing a radial symmetrical distribution from the coronal centre, are generally located next to the site where the semisolid agar was pierced by the inoculating toothpick. Each pillar has a characteristic bush-like morphology typically measuring 600–800 μm and is generated by growth, self-clumping and self-aggregation of cells (Figure 1F-J). The corona requires the rupture of the semisolid agar prior to its formation (Figure 2A-1). Thus, this formation was not observed in those cases for which there was no contact between the toothpick and the plastic, or that developed exclusively on semisolid agar surface (Figure 2A-2) indicating that *E.coli* does not have the ability to drill deeply into 0.6% ABE semisolid agar. Interestingly, it was observed by removing the exterior macrocolony that the thinnest, most superficial layer of semisolid agar was colonized by bacteria (Figure 2F-G).

It is well known that bacterial biofilm formation is affected by the kind of surface on which they develop, with plastic being favoured over glass for biofilm building [16]. Thus, corona formation was completely absent when *E. coli* K-12 MG1655 strain was grown in a Pirex® glass Erlenmeyer (Figure 2E). Therefore, contact with a plastic surface must trigger the corona formation (Figure 2D) which indicates that the surface itself must be sensed for biofilm formation to occur.

On the other hand, it is thought that the hardness of the agar influences the behaviour of *E. coli* bacteria [41]. Thus, *E. coli* is able to swim driven by flagella in low hardness agar (0.25-0.5%) whereas higher agar concentrations (e.g., 1.5%) impede flagella expression and thus suppress *E. coli* motility [41]. Due to the fact that the corona develops inside semisolid agar, we wondered whether this also occurs for corona formation. In order to test this possibility, the ability of *E. coli* to form a corona was assayed in a 1.0% (1 g/L) ABE agar concentration. Unexpectedly, an increase in the hardness of agar from 0.6% to 1.0% did not reduce the size (diameter) of corona (Figure 2H-I), but, in fact, stimulated the ability of *E. coli* to penetrate into semisolid agar, i.e., their spreading and colonizing capabilities.

Additionally, this coronal size increase was accompanied by a change in the morphological appearance of the coronal flares, which become less bushy (Figure 2J-K). Notably, the corona can even overtake and surpass the size of the macrocolony growing on the surface (Figure 2H-I). Residual corona formation was still observed at 1.5% ABE agar concentration (data not shown).

Taking into account the importance of bacterial movement for colony formation, a key issue was to determine whether flagella were involved in the corona formation through the promotion of bacterial spreading in semisolid agar or in pillar formation. To address these questions, *E. coli* strains that were defective in flagellar and chemotaxis genetic networks were tested. All mutant strains maintained the ability to form a full-fledged corona (see Additional file 1A-N, the mutants assayed are detailed in legend of this figure), indicating that bacterial spreading inside semisolid agar and the formation of coronal spikes did not require flagella, the chemotaxis sensory system or the action of the flagellar motor.

However, since flagella-mediated motility is inversely regulated by biofilm formation [22], it could be expected that the FlhDC + σ^70^/σ^F^-dependent expression of flagella would negatively affect corona formation. To test this possibility a toothpick was used to inoculate the MG1655 strain in a LB "swimming" medium prepared with a 0.5% ABE agar. As shown in Figure 2B-C, the bacterial cells are able to migrate inside the agar propelled by flagella and manage to make contact with the plastic surface of the bottom of the Petri dish, but under these motility conditions the corona did not develop. This result indicates that the flagella driven-motility inhibited the corona formation.

Another appendage of the *E. coli* cell surface involved in biofilm formation is type 1 pili which strengthens the bacteria-to-surface interactions [11] and promotes surface motility [42]. A MG1655 Δ*fimA* strain lacking FimA the major subunit of *E. coli* type 1 pili showed normal corona formation (see Additional file 1O-P), indicating that the processes of adhering to plastic surfaces to build coronas and the spread of *E. coli* when it is submerged in semisolid agar are not mediated for this kind of fimbria.

Intriguingly, when the coronal spikes were disaggregated mechanically it was observed that they are apparently embedded in a matrix made up of unknown transparent adhesive substance(s) (Figure 1I-J). Since CsgD controls the expression of curli fimbria [22], we wondered if CsgD could influence the corona formation. To assay this possibility, the ability of the MG1655 Δ*csgD*::*aadA* Spec^r^ and the W3110 Δ*csgD::cat* strains to generate corona compared with their wild-type strains was assayed in 0.6% semisolid agar Both Δ*csgD* mutants lacking of CsgD were not impaired in the development of the corona (see Additional file 1Q-R), indicating that generation of this architecturally complex pattern does not require the activity of CsgD, and additionally suggests that the adhesive matrix that supports the coronal spikes are not formed by curli fibres. Furthermore, *E. coli* K-12 strains do not produce cellulose [13] and a mutant (Δ*rcsB1320*) that lacks the regulator RcsB, required to activate the production of exopolysaccharide colanic acid [28], develops a normal corona (data not shown). Furthermore, an *E. coli* MG1655 Δ*pga*::*Kan* strain mutant (harbouring a deletion of the *pgaA* gene, encoding the porin through which PGA exopolysaccharide is excreted [15]), defective in PGA production, also exhibits normal corona formation (see Additional file 1S). Altogether, these facts suggest the possibility that a novel EPS adhesive substance(s) might be produced specifically during corona formation.

In the σ^S^/MlrA/CsgD cascade, the σ^S^ (RpoS) sigma factor σ^S^ (the master regulator of the stationary phase and general stress response [28]) and the bacterial signalling molecule bis-(3′-5′)-cyclic dimeric guanosine monophosphate (c-di-GMP) control the *csgD* expression [22,28,43]. C-di-GMP is produced and degraded by multiple diguanylate cyclases (DGCs; characterized by GGDEF domains) and phosphodiesterases (PDEs; with EAL domains), respectively [42]. It has been reported that two separate DGC–PDE c-di-GMP control modules (YdaM–YciR and YegE–YhjH) involving GGDEF/EAL proteins which participate in the turnover of c-di-GMP, either by synthesizing (DGC, diguanylate cyclases) or degrading (PDE,phosphodiesterases) c-di-GMP, converge to control the transcription of the curli operons [22,28,43]. *E. coli* mutant strains defective in production of RpoS (see Additional file 1U-V) and of the two separate DGC-PDE systems YdaM–YciR (see Additional file 1T and Y) and YegE–YhjH (see Additional files 1J and 1W) showed a normal corona formation. These results provide additional support for the evidence that the corona is a CsgD-independent and σ^S^-independent biofilm formation.

**Figure 3:**
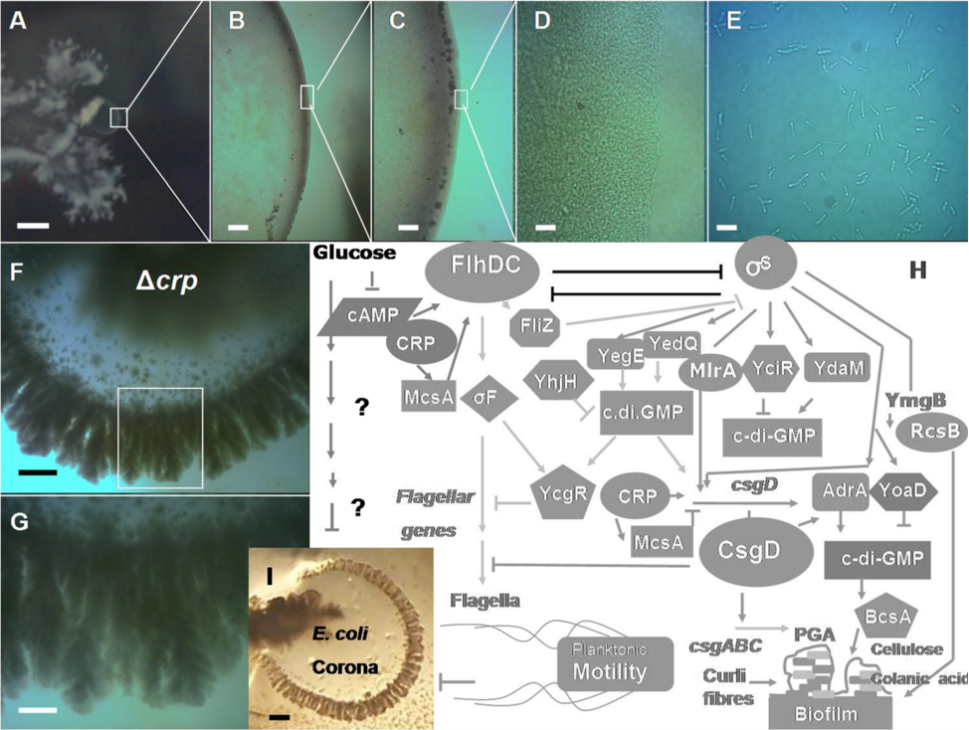
**(A-G) Glucose represses corona formation independently of the regulatory activity of the cAMP-CRP complex. (A)** A typical 14-old-day biofilm macrocolony of *E.coli* K-12 strain grown over 0.6% ABE semisolid agar with Luria Bertani medium supplemented with 0.5% of D-(+)-glucose does not produce a corona **(B-C)** Circular cellular formation surrounding the inoculation point does not produce coronal flares or spikes **(B)** × 40 **(C)** ×100 (**D)** × 400 magnifications **(E)** Individual elongated cells 5–10 μm long, typical "swarm" cells [24,25], removed from macrocolony and observed under optical microscope at × 1000 augmentation **(F-G)** Corona generated by a *E.coli* K-12 Δ*crp* mutant strain (GS0549) lacking CRP protein. (G) Enlargement of the boxed region in F. Scale bars: (A) 0.25 cm (B) 400 μm (C) 200 μm (D) 40 μm (E) 20 μm (F) 350 μm (G) 150 μm. **(H-I)** Corona formation in relation to the canonical "core" transcriptional network that controls switching between motility and biofilm formation. In *E. coli* K-12, the transition from a planktonic/foraging lifestyle to biofilming behaviour is regulated by two inversely controlled transcriptional feedforward cascades, the FlhDC + σ^70^/σ^F^ "flagellar" cascade and the σ^S^/MlrA/CsgD cascade (adapted from references [17,22,28]). Remarkably, while CRP in conjunction with the small RNA McsA has a dual role in the control of both cascades (forming a coherent feedforward loop (FFL) in order to regulate the expression of *flhDC* and an incoherent FFL to control *csgD* expression, [33]) apparently it plays no role in *E. coli’s* corona formation at all. Arrowheads indicate positive regulation; perpendicular lines indicate negative regulation. Scale bar: (I) 800 μm.

Another line of investigation was to determine whether catabolite repression (CR) affected coronal development. To evaluate this possibility, we sought to determine whether D-(+)-glucose could affect the corona formation. Thus, the LB medium was supplemented with 0.5% (5 g/L) of this sugar and the ability of *E. coli* wild-type strains to form coronas was assayed on 0.6% semisolid ABE agar. It was observed that glucose has a strong impact on the morphotypical aspect of the colony. Notably the volcano-like morphotype was disrupted completely, becoming a "softer", more delicate macrocolony form, exhibiting numerous dendritic ramifications (Figure 3A), and although bacteria cells could still generate a circular formation inside the semisolid agar surrounding the inoculation point, the formation of typical coronal spikes was totally abolished (Figure 3B-E). Interestingly, the development of this circular formation was not observed in Δ*fliC*, Δ*flhDC*, Δ*fliA* and *ΔmotA E. coli* K-12 mutant strains (data not shown), indicating that under these nutritional conditions the flagella are required for *E. coli’s* migration inside 0.6% ABE semisolid agar. Additionally, these results suggest that although the flagella could support the bacterial migration at this level of agar concentration, the expression of flagella is probably suppressed during the formation of the corona.

To determine whether the glucose inhibiting effect on the corona formation was subject to classical catabolite repression via cAMP-CRP complex, the effect of Δ*crp* mutation on development of corona was examined. Surprisingly, as can be seen in Figure 3F-G, the corona formation in this mutant is completely normal compared with the wild-type strain, indicating that the effect of glucose in corona formation in not mediated by this transcriptional regulatory complex.

Overall, these experimental results make it possible to discuss the unique and notable characteristics of the *E. coli* crowning behaviour that identify it as a singular biological phenomenon:

(i) Since neither flagella nor the type 1 pili are required for corona formation, the question of how the bacteria spread inside semisolid ABE agar to create this annular pattern must be addressed. This could be the by-product of bacterial growth, but the fact that an increase in the hardness of the agar leads to an increase in the corona size (Figure 2H-I) rules out this possibility, implying that the bacterial spread inside semisolid agar that results in corona formation might depend on hitherto unknown bacterial structure(s) that actively generate bacterial movement under these special environmental conditions.

(ii) The most enigmatic aspect of corona formation is how the coronal spatial architecture is achieved. In other words, how does *E.coli* crowning behaviour actually work? It is well established that *E. coli* is able to swarm outwards through the water-filled channels in plates containing semisolid 0.25-0.3% nutrient agar forming a ringed (circular) concentric band during this migration process [44]. These classic chemotactically induced rings form because of chemotactic responses to spatial gradients generated by transport and metabolism [21,45]. Chemotactic signalling genes known for chemoreception and chemotactic signal processing are required to form these bands [21] and stripes [46]. Intriguingly, because *E.coli* crowning behaviour does not depend on flagella or chemotactic activities, there should be no associated circularly distributed chemotactical signals to guide corona formation. Thus, to our knowledge, crowning represents the first documented self-organized behaviour that allows *E. coli* to generate a characteristic annular pattern without involving chemotaxis. Hence, the corona is a good model for studying pattern formation in non-chemotactically guided environmental conditions [47].

(iii) These observations make it possible to suggest the hypothesis that another *E.coli* signalling system(s) able to respond to a generated spatial-temporal circular signal pattern might participate in the control of crowning behaviour. For instance, in this respect an important point for future research will be to study the possibility that mechanisms responding to population density (e.g., quorum sensing [4,48,49]) might be involved in the control of corona development.

(iv) Another singular aspect of corona formation that distinguishes it from other kinds of *E. coli* biofilm formations is that while the availability of glucose negatively influences biofilm formation via the cAMP-CRP regulatory complex, corona formation, on the contrary, does not require this classical catabolic repression system indicating that the glucose suppressive effect on corona formation must involve other mechanism(s) that are still to be elucidated (Figure 3H).

## Conclusions

In conclusion, we have presented a novel behaviour of *E. coli* called "crowning" which is the ability exhibited by bacteria to form a biofilm corona. Crowning expands *E. coli’s* repertoire of colonizing behaviours allowing it to colonize those ecological niches where flagella cannot perform adequately. Given the notable characteristics of self-organization exhibited by the corona, this formation represents an excellent biofilm model with which to explore the molecular mechanisms underlying the generation of complex 3-D biofilm architectures. It is clear that corona formation requires exquisite spatial and temporal coordination of self-organized bacterial activity. Understanding the complex dynamics of corona formation is a challenge for future experiments. Finally, the newly studied *E. coli* coronal pattern and the associated behaviour that produces it, reminds us of the self-engineering capabilities of bacteria to structure their bacterial cities in order to adapt and survive in changing environmental situations [50].

## Methods

### *Escherichia coli* strains, media and growth conditions

The *E. coli* strains used in this study are detailed in Table 1. The behaviour of each mutant strain in the corona formation process (obtained from different laboratories as is detailed in this table) was compared with its respective wild-type strain. The experiments that allowed us to unveil the *E. coli* volcano-like and corona formations were conducted using the following protocol: cells obtained from a colony of *E. coli* K12 strains (Table 1) grown in Luria-Bertani medium [38]: 10 g/L (1.0%) Difco® Bacto-Trypone, 0.5% (5 g/L) Difco® Yeast Extract and NaCl 5 g/L (0.5%) harnessed with 1.5% (10 g/L) of *Agar Bacteriológico Europeo* (ABE) were inoculated with toothpick at the centre of a 8.5-cm and 4.5 cm Petri dish made of polystyrene plastic (fabricated by Sterilin® Company, http://www.sterilin.co.uk and Sarstedt® Company, http://www.sarstedt.com respectively) containing 30 ml of LB medium jellified with the indicated ABE concentrations. The plates were sealed with parafilm® to prevent loss of water. After 14 days of incubation at 37 °C, the plates were photographed with reflected light with a digital Kodak *EasyShare* Z710 camera. D-(+)-glucose 0.5% (5 g/L) (provided by Merck Company) was added to LB medium when indicated. The microscopic images were taken with a Ultralyt ULNM-90-10000 microscope (made by *Brown & Crown* Company). The images shown in Figure 1C-E were taken with a Leika M205 FA stereoscopic microscope coupled with a DFC350FX digital camera. The images of the figures were framed with *Microsoft Photo Editor software* and composed using the *Powerpoint* software program.

**Table 1:** ***Escherichia coli *** **K-12 strains used in this study**

***E.coli *** **strain**	**Relevant genotype**	**Source or reference**
MG1655	Wild-type*	I. Tagkopoulos [51]
		J. M. Ghigo [13]
		H. Suzuki [42]
		G. Storz [33]
MG1655 ∆*csgD*	∆*csgD*::*aadA* Spec^r^	J. M. Ghigo
NM525	Wild-type*	
GS0548	MG1655 ∆*rpoS*::*kan*	G. Storz
GS0549	MG1655 ∆*crp::cat*	"
GS0551	MG1655 ∆*pgaA*::*kan*	"
GS0553	MG1655 ∆*flhDC*::*kan*	"
GS0554	NM525 ∆*csgD*::*kan*	"
MG1655 Str^r^	Wild-type*	P.S. Cohen [52]
MG1655 Str^r^ ∆*flhD*::*cam*	∆*flhD*::*cam*	"
MG1655 Str^r^ ∆*motBA* ∆*fliC*::*cam*	∆*motBA* ∆*fliC*::*cam*	"
SK650	MG1655 ∆*fimA*::FRT-*kan* + -FTR	H. Suzuki
SK598	MG1655 ∆*fliC*::FRT-*Kan* + -FTR	"
BW25113	Wild-type*	T. Mizuno [53]
BW28079	BW25113 ∆(*flhEAB cheZYBR tap tar cheWA motBA flhCD* IS*1*)*1218*	"
BW27870	BW25113 ∆*rcsB1320*	"
W3110	Wild-type*	R. Hengge [22]
AR120	W3110 ∆*fliC::kan*	"
GB301	W3110 ∆*yhjH::cat*	"
GB303	W3110 ∆*ycgR::kan*^R^	"
GB304	W3110 ∆*csgD::cat*	"
GB328	W3110 ∆*fliA*::*kan*	"
GB331	W3110 *∆motA::kan*	"
GB332	W3110 ∆*flhDC::kan*	"
NS48	W3110 ∆*ydaM::cat*	"
NS49	W3110 ∆*yciR::kan*	"
AR4	W3110 ∆*yegE::kan*	"

Notes: (*), Wild-type to corona formation.

## Abbreviations

ABE: Agar Bacteriológico Europero; cAMP: Adenosine 3′,5′-cyclic monophosphate; EPS: Extracellular polymeric substances; c-di-GMP: bis-(3′-5′)-cyclic dimeric guanosine monophosphate; CRP: cAMP receptor protein; CR: Catabolic repression; DGC: Diguanylate cyclases; LB: Luria-Bertani medium; PDE: Phosphodiesterases; PGA: β-1,6-*N*-acetyl-D-glucosamine polymer.

## Competing interests

The authors declare that they have no competing interests.

## Authors’ contributions

JMGG and RA participated in the design of the study as well as drafted, developed and wrote the entire manuscript. JMGG performed the first phenotypic observation and carried out the phenotypic and genetic studies. Both authors read and approved the final manuscript.

## Supplementary Material

Additional file 1 **Corona generated by different *E. coli *** **K-12 strain mutants. (A-C)***∆flhDC* strain lacking of flagellar master regulator FlhDC **(A-B)** ∆*flhD*::*cam***(C)** ∆*flhDC*::*kan***(D)** ∆*fliA* strain lacking of flagellar alternative sigma, σ^F^ (RpoF) **(E-F)** ∆*motAB* ∆*fliC* strain defective in the production of the basic subunit of a flagella filament flagellin FliC and MotAB, the stator flagellar rotor **(G-H)** ∆*motA***(I)** ∆*fliC***(J)** ∆*yhjH***(K-L)** ∆*cheZYBR* ∆*cheWA* lacking of two component CheA/BCheY chemotaxis signalling system **(M-N)** ∆*ycgR***(O-P)** ∆*fimA***(Q-R)** ∆*csgD***(S)** ∆*pgaA***(T)** ∆*ydaM***(U-V)** ∆*rpoS***(W)** ∆*yegE***(Y)** ∆*yciR*. Magnifications and scale bars: (A, C, D, E, G, K, M, O, Q, S, T, U) × 40, 200 μm; (B, F, H, I, J, L, N, P, R, V, W, Y) × 100, 100 μm. Click here for file
