# Supplementary figures and images for: Crowning: a novel Escherichia coli colonizing behaviour generating a self-organized corona
Source: BMC Res Notes. 2014 Feb 25;7:108. doi: 10.1186/1756-0500-7-108 (PMC3936827; doi:10.1186/1756-0500-7-108)

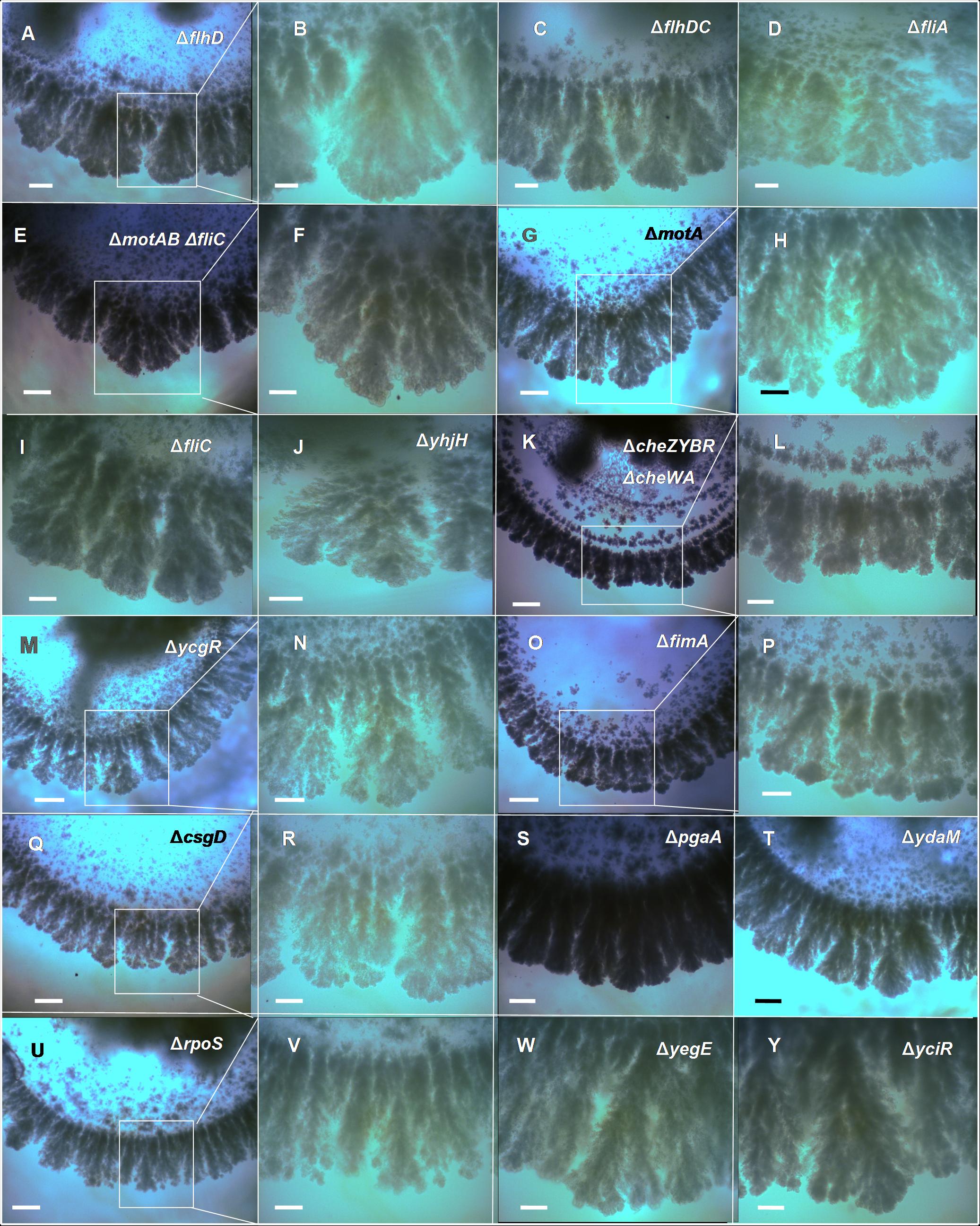

Supplement: Additional file 1 — Corona generated by different E. coli K-12 strain mutants. (A-C)∆flhDC strain lacking of flagellar master regulator FlhDC (A-B) ∆flhD::cam(C) ∆flhDC::kan(D) ∆fliA strain lacking of flagellar alternative sigma, σF (RpoF) (E-F) ∆motAB ∆fliC strain defective in the production of the basic subunit of a flagella filament flagellin FliC and MotAB, the stator flagellar rotor (G-H) ∆motA(I) ∆fliC(J) ∆yhjH(K-L) ∆cheZYBR ∆cheWA lacking of two component CheA/BCheY chemotaxis signalling system (M-N) ∆ycgR(O-P) ∆fimA(Q-R) ∆csgD(S) ∆pgaA(T) ∆ydaM(U-V) ∆rpoS(W) ∆yegE(Y) ∆yciR. Magnifications and scale bars: (A, C, D, E, G, K, M, O, Q, S, T, U) × 40, 200 μm; (B, F, H, I, J, L, N, P, R, V, W, Y) × 100, 100 μm. [file 1756-0500-7-108-S1.jpeg]
